# A novel combination of four flavonoids derived from Astragali Radix relieves the symptoms of cyclophosphamide‐induced anemic rats

**DOI:** 10.1002/2211-5463.12146

**Published:** 2017-01-28

**Authors:** Li Zhang, Amy G. W. Gong, Kashif Riaz, Jun Y. Deng, Chih M. Ho, Huang Q. Lin, Tina T. X. Dong, Yi‐Kuen Lee, Karl W. K. Tsim

**Affiliations:** ^1^ Division of Life Science and Center for Chinese MedicineThe Hong Kong University of Science and TechnologyChina; ^2^ School of PharmacyShanghai University of Traditional Chinese MedicineChina; ^3^ Department of Mechanical and Aerospace EngineeringThe Hong Kong University of Science and TechnologyChina; ^4^ Department of Mechanical and Aerospace EngineeringUniversity of CaliforniaLos AngelesCAUSA; ^5^ HKUST Shenzhen Research InstituteNanshanShenzhenGuangdong ProvinceChina

**Keywords:** feedback system control, flavonoids combination, hematological parameters

## Abstract

By using a feedback system control scheme, the best combination of formononetin, ononin, calycosin, and calycosin‐7‐*O*‐β‐d‐glucoside derived from Astragali Radix was shown to activate a hypoxia response element, a regulator for erythropoietin (EPO) transcription, in kidney fibroblast. In cyclophosphamide‐induced anemic rats, the treatment of combined flavonoids, or EPO, improved the levels of red blood cells, white blood cells, hemoglobin, and hematocrit. In addition, the altered levels of antioxidant capacity, super oxidase dismutase, and malondialdehyde, triggered in anemic rats, were restored to control levels by the treatment of flavonoids. Here, we proposed a possible therapy by using the common flavonoids in treating anemia.

**Abbreviations**
CF: combined flavonoids
CYP: cyclophosphamide
DE: differential evolution
EPO: erythropoietin
ESA: erythropoiesis‐stimulating agents
FSC: feedback system control
HIF: hypoxia‐induced factor
HRE: hypoxia responsive element
MDA: malondialdehyde
SOD: super oxidase dismutase
T‐AOC: total antioxidant capacity
TCM: traditional Chinese medicine

Anemia is a blood disorder associated with a series of health problems, for example, chronic kidney disease, congestive heart failure, chronic renal failure, diabetic, and nondiabetic renal disease 1. In addition, the treatment of cancer in combining radiotherapy, chemotherapy, and noncardiac or nonvascular surgery could lead to anemia 2, 3. Usually, blood transfusion is required for the anemic patients before therapy and surgery of these illnesses 4. Nevertheless, blood transfusion should be avoided wherever possible because of high risk of infection, mortality, and deaths 5, 6.

Erythropoiesis‐stimulating agents (ESA), that is, recombinant human epoein‐α and darbepoetin‐α, have been utilized for over two decades for the treatment of anemia associated with various diseases, in particular, cancer therapy‐induced anemia 7, 8. In clinical studies, the usage of ESAs in patients could cause a high risk of mortality, thromboembolic event, tumor progression, myocardial infarction, stroke, and hypertension 9, 10. Cyclophosphamide (CYP), a nitrogen mustard alkylating agent, is used in the treatment of cancer chemotherapy and autoimmune disorders 11, 12. In clinical application, CYP exhibits many side effects, including bone marrow suppression and cytotoxicity leading to progressive anemia, oxidative stress, and immunological suppression 13, 14.

Due to its low toxicity, traditional Chinese medicine (TCM) has a long history of utilization as complementary health food supplements 15. Historically, Astragali Radix (roots of *Astragalus membranaceus* (Fisch.) Bge. var. *mongholicus* (Bge.) Hsiao or *A. membranaceus* (Fisch.) Bge.) is considered as major herb in stimulating ‘Qi’. Pharmacological study of Astragali Radix has shown that it could enhance biological functions, such as hepatoprotective effects, neuroprotective functions, hematopoietic, antioxidative, immunological properties, and antiaging activities 16, 17. The major ingredients within Astragali Radix, that is, formononetin, ononin, calycosin, and calycosin‐7‐*O*‐β‐d‐glucoside, have been employed to enhance hematopoietic functions by regulating erythropoietin (EPO) expression; hence, possibly could be used in treating anemia 18, 19, 20. TCM employs a combinatorial therapeutic approach: this is essential to identify an optimal drug cocktail in terms of efficacy and low side effects. This is a challenge due to the large number of possible combinations of such herbal mixtures. Recently, an engineering approach, namely feedback system control (FSC) scheme, has been developed to search potent drug combinations in tens of iterations out of a hundred thousand possible trials 21, 22. By using this novel FSC screening method, we found the best combination of formononetin, ononin, calycosin, and calycosin‐7‐*O*‐β‐d‐glucoside in activating the transcriptional activity of EPO gene 20. To verify the usage of combined flavonoids, CYP‐induced anemic rats were treated with the flavonoids. The serum parameters of hematology, oxidation and immunology were systematically compared in CYP‐induced rats.

## Materials and methods

### Materials and chemicals

Formononetin, ononin, calycosin, and calycosin‐7‐*O*‐β‐d‐glucoside were purchased from Weikeqi Biotechnology Co. (Sichuan, China). HPLC was employed to determine the purities of these marker chemicals, which showed over 98.0% of purity. Analytical‐ and HPLC‐grade reagents were purchased from Merck (Darmstadt, Germany).

### Optimization of flavonoid combination

The FSC scheme was performed by an iterative closed‐loop of three processes which includes drug combination preparation, experimental readout, and search algorithm. To determine the optimal flavonoid combination, the maximal transcriptional activity of EPO gene in cultured human embryonic kidney fibroblast (HEK 293) was measured by using luciferase assay 20. The readouts from luciferase assay were fed into stochastic search algorithm (DE, differential evolution), which used the readout as fitness index and generated the new flavonoid combination for next luciferase assay. This process was repeated until stable optimal flavonoid combination was found. The optimal combination 0.08, 0.08, 0.4, and 0.08 μm (a ratio of 1 : 1 : 5 : 1) of four flavonoids, that is, formononetin, ononin, calycosin, and calycosin‐7‐*O*‐β‐d‐glucoside, was established that demonstrated ~ 3‐fold increase in transcriptional activity as compared to control 20. The same ratio of combined flavonoids was used for animal experiments.

### Animals and experimental design

Male Sprague–Dawley rats (4 weeks old) weighing 180–220 g were provided by Laboratory Animal Services Center, China Pharmaceutical University (Nanjing, China). The rats were housed under controlled environmental conditions of temperature (25 ± 2 °C) and 12 h of light and dark cycle. All animal‐handling procedures were performed in strict accordance with China Legislation on The Use and Care of Laboratory Animals, with the guidelines established by Institute for Experimental Animal of China Pharmaceutical University, and which were approved by the college committee for animal experiments. Rats were divided into five major groups, each group contained eight rats: normal (treated with saline only), control (treated with CYP), EPO (treated with CYP + EPO), CF (treated with CYP + combined flavonoids at high and low doses). All rats were first intraperitoneally injected with CYP (25 mg·kg^−1^) for 4 days to induce anemia, except the normal rats. After 4 days, the control group was treated with saline, EPO group was intraperitoneally injected with EPO (75 IU·kg^−1^·day^−1^), the flavonoid group was intraperitoneally injected with a low dosage of flavonoid combination (11.2 μmol·kg^−1^·day^−1^) or with a high dosage of flavonoid combination (32 μmol·kg^−1^·day^−1^), for 14 days. The flavonoid mixture (32 μmol), containing 4 μmol formononetin, 4 μmol ononin, 20 μmol calycosin, and 4 μmol calycosin‐7‐*O*‐β‐d‐glucoside, that is, 1 : 1 : 5 : 1 ratio, was dissolved in saline.

### Biochemical measurement

Twenty‐four hours after the last injection, the blood samples were collected from the eye pit, centrifuged at 3000 ***g*** for 15 min at 4 °C, so as to obtain serum for hematopoietic function test. Then, all rats were weighed and sacrificed by cervical dislocation; spleen, liver, kidney, and thymus were removed immediately and weighed. Super oxidase dismutase (SOD), malondialdehyde (MDA) and total antioxidant capacity (T‐AOC) were determined spectrophotometrically according to the instructions for commercial kits, while serum IL‐2 level was determined by ELISA using commercially available kits (Jiancheng Bioengineering Institute, Nanjing, China). Red blood count, white blood count, hemoglobin, and hematocrit levels were revealed according to the commercial instructions for the automatic biochemical analyzer (Biochemical Analytic Center of Zhongda Hospital, Nanjing, China).

### Statistical analysis

The data are expressed as mean ± SD value, *n* = 6–8. Statistical tests were performed by two‐way ANOVA. Statistically significant changes were classed as [*] where *P* < 0.05; [**] where *P* < 0.01 and; [***] where *P* < 0.001.

## Results

**Figure 1:**
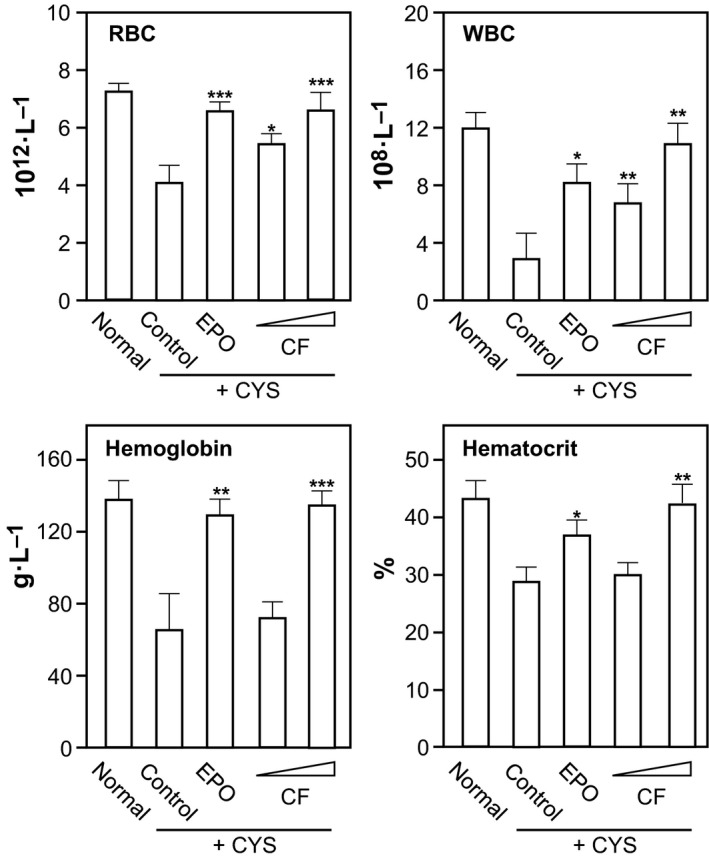
Combined flavonoids on hematological parameters in anemic rats. Rats were injected with sterile water or with CYP (25 mg·kg^−1^) for 4 days. Then, the rats were divided into five groups. Normal: normal rats treated with sterile water (normal); Control: CYP‐treated rats with saline; EPO: CYP‐treated rats with EPO (75 IU·kg^−1^·day^−1^ body weight); CF: CYP‐treated rats with low dose (11.2 μmol·kg^−1^·day^−1^) or with a high dose of flavonoid combination (32 μmol·kg^−1^·day^−1^). The flavonoid mixture (32 μmol), containing 4 μmol formononetin, 4 μmol ononin, 20 μmol calycosin, and 4 μmol calycosin‐7‐*O*‐β‐d‐glucoside, was dissolved in saline. The number of red blood cell (RBC), white blood cell (WBC), amount of hemoglobin, and the percentage of hematocrit were determined. Data are expressed as Mean ± SD, where *n* = 6–8. [*] where *P* < 0.05; [**] where *P* < 0.01 and; [***] where *P* < 0.001 as compared with control group.

Before performing the biological assay, the weight index of spleen, liver, kidney, and thymus did not change significantly, as shown in Table S1. The blood hematological parameters in anemic rats are shown in Fig. 1. The levels of red blood cells, white blood cells, hemoglobin, and hematocrit were significantly decreased in CYP‐induced anemic rats, indicating that CYP injection successfully induced anemia in rats. The anemic rats were treated with EPO and combined flavonoids for 14 days. In both cases, the hematological parameters of anemic rats were restored to normal level (Fig. 1). High dose of combined flavonoids (32 μmol·kg^−1^·day^−1^) showed better effects, which fully restored the reduced hematological parameters in anemic rats, that is, 6.37 ± 0.73 × 10^12^ L^−1^ in red blood count, 11.85 ± 1.94 × 10^9^ L^−1^ in white blood count, 127.01 ± 0.93 g·L^−1^ in hemoglobin, and 41.72 ± 3.69% in hematocrit count (Fig. 1). These parameters were very similar to the EPO‐treated anemic rats, except the white blood count, which was much better in the scenario of flavonoid treatment. These parameters suggested that the therapeutic effect of a novel flavonoid mixture could replace the function of EPO in anemic rats.

**Figure 2:**
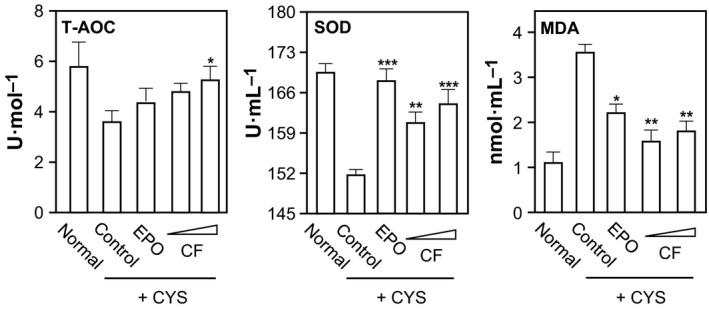
Combined flavonoids on antioxidative properties in anemic rats. The treatment of anemic rats, as well as the grouping, was carried out as in Fig. 1. The levels of antioxidant capacity (T‐AOC), super oxidase dismutase (SOD), and malondialdehyde (MDA) were measured in the rat serum. Data are expressed as Mean ± SD, where *n* = 6–8. [*] where *P* < 0.05; [**] where *P* < 0.01 and; [***] where *P* < 0.001 as compared with control group.

The nonenzymatic antioxidant defense system capacity is reflected by T‐AOC. In the anemic rat model, CYP dramatically decreased markedly the serum levels of T‐AOC and SOD. In contrast, the injection of CYP increased the level of MDA (Fig. 2). After the administration of combined flavonoids, the altered levels of T‐AOC, SOD, and MDA in rat serum were restored back to normal. Again, a high dose of combined flavonoids showed a robust effect (Fig. 2). EPO showed similar effects to that of the flavonoids in restoring the parameters back to normal.

**Figure 3:**
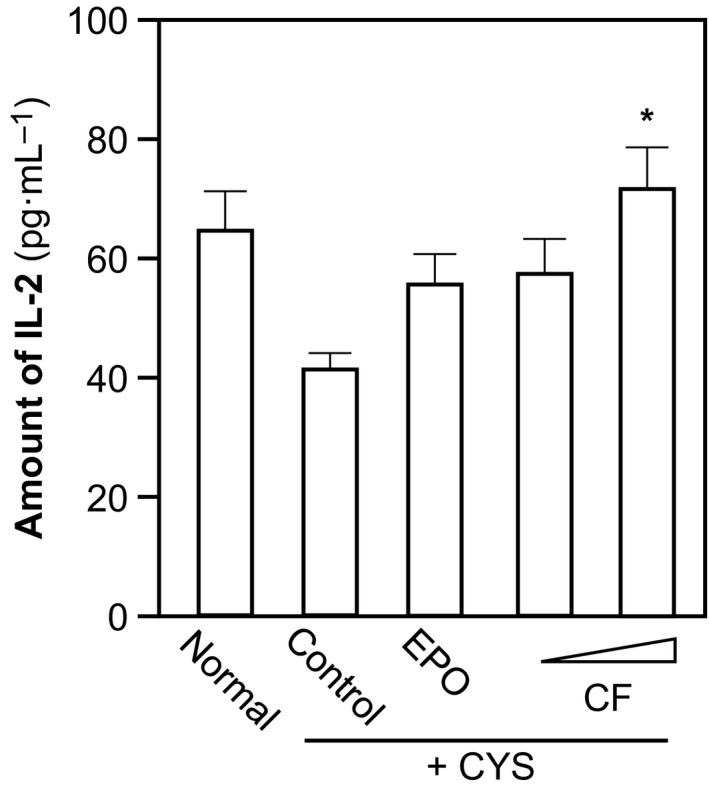
Combined flavonoids on serum level of IL‐2. The treatment of anemic rats, as well as the grouping, was carried out as in Fig. 1. The amount of IL‐2 was measured by ELISA. Data are expressed as Mean ± SD, where *n* = 6–8. [*] where *P* < 0.05 as compared with control group.

Long‐term administration of CYP could result in immunosuppression, that is, because of the deficiency of EPO. Interleukin (IL)‐2, secreted by T lymphocytes, is an important factor to promote immune cell proliferation and differentiation 23. A significant decrease (~ 26%) in IL‐2 was revealed in the rat serum after CYP administration (Fig. 3). The administration of combined flavonoids, or EPO, restored the reduced level of IL‐2 back to normal (Fig. 3). High dose of flavonoids showed a significantly better effect, as compared to EPO control. Our results clearly showed that flavonoids not only recovered the hematological parameters but also possessed oxidant inhibition and immunological enhancement activities.

## Discussion

Traditional Chinese medicine is a style of traditional Asian medicine building on a foundation of more than 2500 years of Chinese medical practice, which includes various forms of herbal medicine, acupuncture, massage, exercise, and dietary therapy. Astragali Radix is the most common herbal medicine on the market. Four major flavonoids, derived from Astragali Radix, formononetin, ononin, calycosin, and calycosin‐7‐*O*‐β‐d‐glucoside, have been shown to be effective in regulating EPO expression, which in turn might improve hematopoietic functions 18, 19. The low levels of these flavonoids being used here could not fully account for the induction activity of EPO, suggesting a possible synergistic interaction among the four flavonoids. Different experimental approaches have been used for the optimization of combinatorial therapies. But these methods require numerous experiments, additional cost, and are time consuming. FSC scheme has been introduced to search for optimized drug combinations using iterative stochastic search in terms of iterations out of a hundred thousand possible trials 21, 22. By using cell culture as a model, we have adopted this novel methodology in optimizing the four flavonoids in combination, which shows strong activation on transcriptional activity of hypoxia responsive element 20.

We have utilized DE as a stochastic search algorithm in FSC scheme. We started with an initial flavonoid combination and measured maximal transcriptional activity of EPO gene in cultured HEK293 using luciferase assay. The results from this experiment were fed back into the algorithm, which used these as fitness index to generate a new flavonoid combination for the next step. These three steps were repeated until we explored a stable optimal flavonoid combination with the highest efficacy and the lowest toxicity 20.

The cytotoxicity of CYP is known due to its reaction with DNA, resulting in CYP‐specific DNA adducts. These adducts are responsible for the formation of interstrand crosslinks, blocking DNA replication and causing bone marrow suppression. Experimental results indicated that there was a decrease in the red blood count, white blood count, hemoglobin, and hematocrit levels after CYP administration. Liver and kidney are responsible for the production of EPO that is an erythrocyte‐specific hematopoietic growth factor 24. A critical regulator for EPO transcription is a hypoxia responsive element, the promoter region of EPO gene 19. EPO expression is initiated when the activated hypoxia‐induced factor (HIF) is being bound onto the DNA promoter 25. These four flavonoids could induce the expression of EPO and therefore, enhance hematological parameters 26. Optimized flavonoid combination after CYP injection showed that hematological parameters have recovered to normal.

Free radical formation is associated with inflammatory and cardiovascular diseases 27. Different organisms have defense systems or detoxification enzymes i.e. SOD and glutathione, to protect us from radical damages 27. Due to the production of free radicals, CYP causes cytotoxicity and oxidative stress 28. SOD is a key enzyme that helps in the clearance of free radicals 29. Here, the injection of flavonoids could increase the activity of SOD in CYP‐treated rats. These data indicated that the combined flavonoids may play a vital role in protecting immune organs from damage by inhibiting the CYP‐induced oxidative stress.

Optimizing the combination of these four flavonoids by the FSC scheme could provide hints in the study of TCM formulae. According to TCM theory, the herbal formulae should be prepared in a unique methodology having a specific combination of different herbs (named as *Fu Fang*). The combination among different herbs within a decoction will directly affect the pharmacological properties of a herbal formula. Indeed, our previous work has supported the usage of the best combination of mixed herbs in an ancient herbal decoction. Thus, the FSC scheme could be a promising method to optimize the combination of a herbal formula, which accelerates the exploration of the veil of TCM.

## Author contributions

LZ and AG conceived, designed and did experiments. KR and JYD performed FSC calculating, CMH, HQL, TTD, YKL and KWT were responsible for drafting the manuscript. All authors reviewed the results and approved the final version of the manuscript.

## Supporting information


**Table S1.** Effects of flavonoids on liver, kidney, spleen, and thymus index.
