# Supplementary material for: A novel combination of four flavonoids derived from Astragali Radix relieves the symptoms of cyclophosphamide‐induced anemic rats
Source: FEBS Open Bio. 2017 Jan 28;7(3):318–23. doi: 10.1002/2211-5463.12146 (PMC5337903; doi:10.1002/2211-5463.12146)
Supplement: Supplementary file 1 — Table S1. Effects of flavonoids on liver, kidney, spleen, and thymus index. [file FEB4-7-318-s001.docx]

**Supplementary Table. Effects of flavonoids on liver, kidney, spleen and thymus index**

| **Group**^a^ | Liver (%)^b^ | Kidney (%) | Spleen (%) | Thymus (%) |
| --- | --- | --- | --- | --- |
| Normal | 3.33^c^ | 0.43 | 0.40 | 0.37 |
| Control | 3.37 | 0.40 | 0.25 | 0.24 |
| EPO | 3.72 | 0.43 | 0.32 | 0.33 |
| CF-L | 3.47 | 0.43 | 0.29 | 0.28 |
| CF-H | 3.15 | 0.39 | 0.30 | 0.28 |

**^a^** Normal: normal rats treated with sterile water; Control: CYP-treated rats with saline; EPO: CYP-treated rats with EPO (75 IU/kg/day body weight); CF-L: CYP-treated rats with low dose (11.2 µmol/kg/day); CF-H: CYP-treated rats with high dosage of combined flavonoids CYP-treated rats with high dose flavonoid combination (32 µmol/kg/day). In 32 µmol of flavonoid mixture, it should contain 4 µmol formononetin, 4 µmol ononin, 20 µmol calycosin, and 4 µmol calycosin-7-O-β-D-glucoside, and which was dissolved in saline.

**^b^** Organ index = the weight of the organs (g)/ the weight of whole body (g)

**^c^** Data are expressed as Mean ± SD of 6 - 8 rats. SD is less than 10% of the Mean, not shown for clarity.
